# Neutrophil to lymphocyte ratio predicts persistent organ failure and in-hospital mortality in an Asian Chinese population of acute pancreatitis

**DOI:** 10.1097/MD.0000000000004746

**Published:** 2016-09-16

**Authors:** Yushun Zhang, Wei Wu, Liming Dong, Chong Yang, Ping Fan, Heshui Wu

**Affiliations:** a Pancreatic Disease Institute, Union Hospital, Tongji Medical College, Huazhong University of Science and Technology, Wuhan, Hubei province; b Shanxi Medical University, Taiyuan, Shanxi province; c Organ Transplantation Center, Hospital of the University of Electronic Science and Technology of China and Sichuan Provincial People's Hospital, Chengdu, People's Republic of China.

**Keywords:** acute pancreatitis, intensive care, mortality, neutrophil to lymphocyte ratio, persistent organ failure

## Abstract

Neutrophil to lymphocyte ratio (NLR) has frequently been reported as a significant indicator of systemic inflammation in various medical conditions. The association underlying NLR and outcomes in patients with acute pancreatitis (AP) has not been evaluated after the publication of revised Atlanta classification.

This was a single-center retrospective diagnostic accuracy study and a cohort outcome study. From 2009 to 2015, Asian Chinese patients with a diagnosis of AP presented within 72 hours from symptom onset and underwent neutrophil, lymphocyte assessment at presentation were included in this study. The outcomes were the occurrence of persistent organ failure (POF), intensive care unit (ICU) stay >7 days, and in-hospital mortality. The relationships of baseline neutrophil, lymphocyte count, and NLR with outcomes were assessed with multivariate Cox regression model.

A total of 974 consecutive AP patients were clinically eligible. The mean neutrophils, lymphocytes, and NLR for the entire population were 10.23 ± 4.76  × 10^9^/L, 1.05 ± 0.49  × 10^9^/L, and 12.88 ± 11.25. Overall, 223 (22.9%) of the patients developed with POF, 202 (20.7%) spent more than 7 days in ICU, and 58 (6.0%) died during hospitalization. The NLR had a superior predictive performance than neutrophils and lymphocytes. Using an NLR cutoff of 11, the area under the curves (AUC) were 0.76 for POF, 0.74 for longer ICU stay, and 0.79 for death during hospitalization. After multivariate analysis, NLR ≥ 11 was further identified as an independent prognostic factor (hazard ratio [HR] 1.37, 95% confidence interval [CI] 1.00–1.89; HR 1.44, 95% CI 1.03–2.00; HR 2.75, 95% CI 1.12–6.76; all *P* value < 0.05). Following stratification according to quartiles of NLR, positive trends for the association across increasing NLR quartiles and the 3 outcomes were observed (*P* values for trends across quartiles were 0.007, 0.016, and 0.028, respectively). The adjusted HRs for highest NLR quartile versus the lowest were 2.80 (95% CI 1.42–5.51) (POF), 2.79 (95% CI 1.37–5.70) (ICU > 7 days), and 2.22 (95% CI 0.49–10.05) (mortality), respectively.

Our data show for the first time that an increased NLR is an independent risk factor for POF, longer ICU stay, and in-hospital mortality in AP.

## Abstract 2

Supplemental Digital Content is available in the text

## 1 Introduction

Acute pancreatitis (AP) represents a paradigm model characterized by local and systemic inflammation, which is observed clinically from no systemic signs through the systemic inflammatory response syndrome (SIRS), organ failure (OF), persistent organ failure (POF), and death.^[1,2]^ The underlying pathophysiology through which local pancreatic injury drives the systemic inflammatory response has not been fully elucidated, but cumulative data suggest that both innate immune system (including neutrophils, monocytes, and macrophages) and adaptive immune system (mainly composed of lymphocytes) play pivotal roles.^[3,4]^

Over the past 1 decade, numerous research groups have investigated the value of the hematological components of the systemic inflammatory response specifically for use in predicting disease severity as well as outcome, and have reported that the individual components of the differential leukocyte count may have clinically predictive utility. Neutrophil to lymphocyte ratio (NLR), a simple, easily calculated systemic inflammation-based score, has been generally investigated in a variety of disease states, including inflammatory, cardiovascular, and neoplastic conditions.^[5–7]^

NLR has been shown an association with disease severity and adverse outcomes in AP.^[8,9]^ However, no study has investigated any possible relationship between NLR and incidence of POF, longer stay in intensive care unit (ICU), and in-hospital mortality in a large population of AP. In the present study, we aimed to evaluate whether there is an association between NLR and the 3 outcomes in patients with AP.

## 2 Materials and methods

### 2.1 Study population

**Figure 1:**
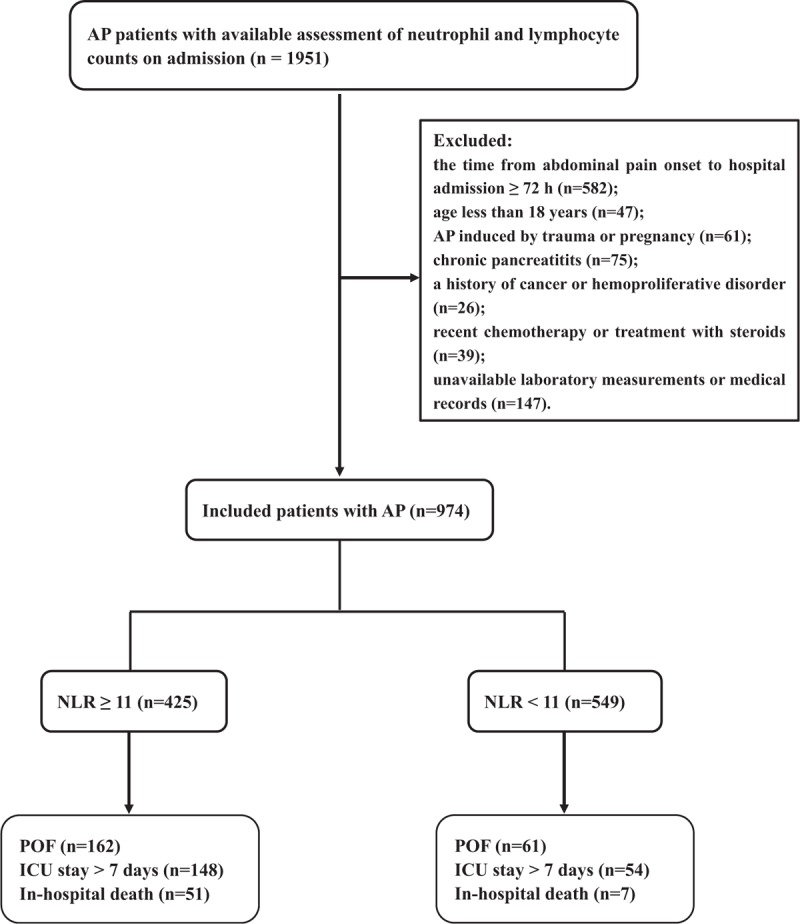
Flow diagram illustrating the selection of patients. AP = acute pancreatitis, ICU = intensive care unit, NLR = neutrophil to lymphocyte ratio, POF = persistent organ failure.

Consecutive patients who admitted to the Pancreatic Disease Institute of Union Hospital (Wuhan, China) with a confirmed diagnosis of AP between January 2009 and January 2015 were included in this retrospective study. AP was defined as clinical findings based on the presence of 2 or more of the following 3 criteria: abdominal pain consistent with AP; serum amylase and/or lipase elevation ≥ 3 times the upper limit of normal; and/or contrast-enhanced computed tomography, magnetic resonance imaging, or abdominal ultrasonography findings characteristic of AP.^[1]^ Of these, 1951 AP patients had laboratory data for neutrophil and lymphocyte counts measured upon presentation to hospital. For this study, individuals were excluded if they met any of the following: the time from abdominal pain onset to hospital admission ≥72 hours (n = 582), age younger than 18 years (n = 47), pancreatitis induced by trauma or pregnancy (n = 61), chronic pancreatitits (n = 93), a history of cancer or hemoproliferative disorder (n = 26), recent chemotherapy or treatment with steroids (n = 39), and unavailable laboratory measurements or medical records (n = 174). As some individuals met more than 1 exclusion criteria, the total number of eligible patients for the study was 974 (Fig. 1).

The study protocol conformed to the ethical guidelines of the 1975 Declaration of Helsinki. The local Ethics Committee of Union Hospital (Wuhan, China) approved this study. Informed consent for individual was exempted because personal identifiable information was removed prior to accession.

### 2.2 Data collection

Electronic medical records and paper charts were reviewed by 1 independent physician for information on demographics (age, sex, and etiology), medical history, smoking habit, alcohol consumption, preexisting comorbidity, and outcome (POF, ICU stay, and in-hospital mortality). Blood samples were collected within 2 hours after hospitalization and analyzed using an automated clinical chemical analyzer within 6 hours of sampling in the same core clinical laboratory in Union Hospital (Wuhan, China).

Neutropenia was defined as a neutrophil count of less than 1.5 × 10^9^/L and neutrophilia as a count of more than 7.0 × 10^9^/L. Lymphocytopenia was defined as a lymphocyte count of less than 1.0 × 10^9^/L and lymphocytosis as a count more than 4.0 × 10^9^/L. The NLR was defined as the division of the absolute neutrophil count to the absolute lymphocyte count measured upon admission to hospital as described previously.^[10]^

### 2.3 Outcomes

The outcomes were incidence of POF, ICU stay > 7 days, and in-hospital mortality. OF was diagnosed according to the modified Marshall score^[1]^ when the following cutoffs were exceeded: cardiovascular failure if systolic blood pressure was <90 mm Hg despite fluid replacement; respiratory failure if the ratio of PaO_2_/FiO_2_ was <300 mm Hg; and renal failure if serum creatinine was ≥1.9 mg/dL. POF was identified if OF lasts for more than 48 hours. The outcome information was centrally adjudicated, in accordance with above definitions, by trained clinicians and radiologists who were blinded to this study.

### 2.4 Statistical analysis

Statistical analysis was performed using SPSS 20.0 (SPSS Inc, Chicago IL). Continuous data are presented as mean and standard deviation. Categorical data are reported as percentages. Student *t* test and Mann–Whitney *U* test were used to evaluate the differences of baseline characteristics between the study cohort and the control group. Multiple group comparisons were performed using the Chi-square test for categorical variables and the Kruskal–Wallis test for continuous data. The diagnostic performance of neutrophils, lymphocytes, and NLR were assessed by receiver operating characteristic (ROC) curves, and the area under the ROC curve (area under the curve, [AUC]) was estimated. Sensitivity, specificity, positive predictive value (PPV), and negative predictive value (NPV) were assessed with 95% confidence interval (95% CI). Univariable, age and sex adjusted, or multivariable analyses for outcomes were performed using a logistic regression model adjusted for potential confounding factors. The confounding covariates included age, sex, smoking habit, alcohol use, preexisting comorbidities such as chronic respiratory, renal, and cardiovascular disease, admission laboratory data, and severity scores. Hazard ratios (HRs) and 95% CIs are presented. *P* values were 2-sided, and a *P* value <0.05 was considered to be statistically significant.

NLR quartiles: quartile 1, <6.25; quartile 2, 6.25 to 9.63; quartile 3, 9.63 to 15.86; and quartile 4, ≥15.86. Neutrophil quartiles: quartile 1, <6.89  × 10^9^/L; quartile 2, 6.89 to 9.60  × 10^9^/L; quartile 3, 9.60 to 12.67  × 10^9^/L; and quartile 4, ≥12.67  × 10^9^/L. Lymphocyte quartiles: quartile 1, <0.70  × 10^9^/L; quartile 2, 0.70 to 0.94  × 10^9^/L; quartile 3, 0.94 to 1.31  × 10^9^/L; and quartile 4, ≥1.31  × 10^9^/L. For testing linear risk trends, we used the quartile rank as a continuous variable in the regression models. We checked the proportional HRs by examining graphs of estimated log (−log) survival.

## 3 Results

### 3.1 Patient characteristics

Baseline characteristics of 974 patients with AP are presented in Table 1. The mean age of the population was 46 years and 561 (57.6%) of the patients were male. The most common cause was biliary-induced AP (56.1%), followed by alcohol-induced AP (22.4%) and hyperlipidemia-induced AP (14.6%), and 6.9% of the patients with unknown etiology. The mean neutrophil count, lymphocyte count, and NLR upon admission for the entire population were 10.23 ± 4.76  × 10^9^/L, 1.05 ± 0.49  × 10^9^/L, and 12.88 ± 11.25, respectively.

**Table 1:**
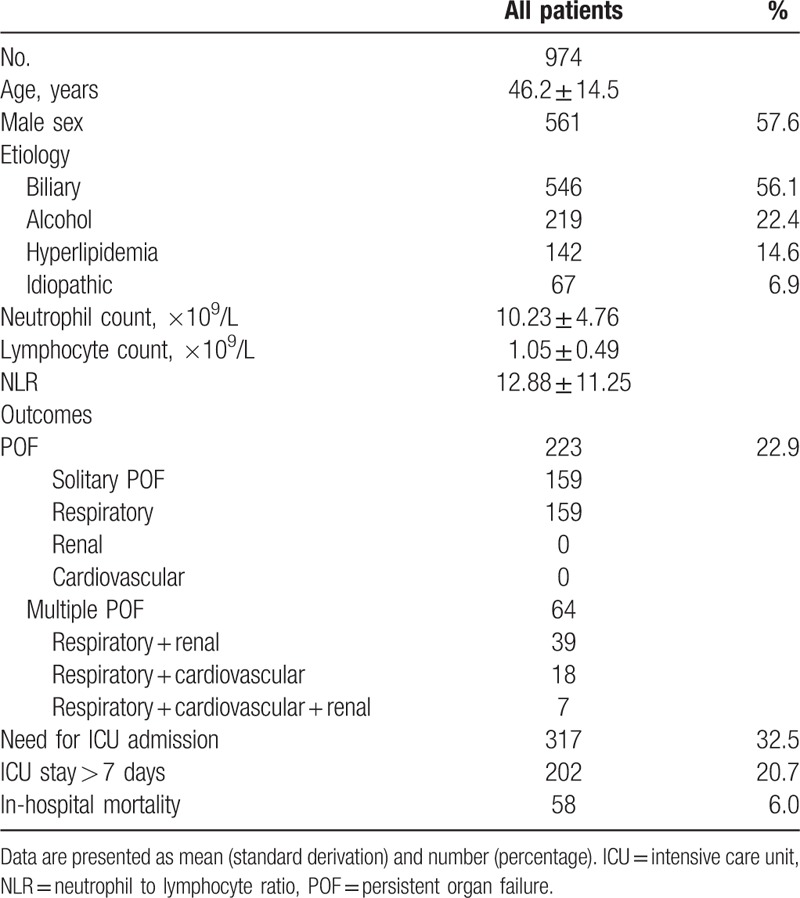
Baseline characteristics of the patients with acute pancreatitis.

### 3.2 Outcomes

Overall, 465 (47.7%) of the patients developed OF. A total of 459 patients developed with pulmonary failure, 67 with renal failure, and 46 with cardiac failure. A total of 223 patients were diagnosed with POF. There were 159 patients developing solitary POF (all in respiratory system). Multiple POF was observed in 64 patients (39 of lung and kidney, 18 of lung and heart, and 7 of lung, kidney, and heart). Accordingly, 317 patients were admitted to ICU, and 202 of them spent more than 7 days in ICU. During hospitalization, 58 patients with POF died with an overall mortality of 6.0%. No death was observed in patients without POF. Fourteen patients died due to persistent respiratory failure, 31 due to multiple POF, and 13 due to infected pancreatic necrosis.

### 3.3 Neutrophil, lymphocyte, and NLR levels on presentation

Mean neutrophil count, lymphocyte count, and NLR at presentation were 12.81 ± 5.57  × 10^9^/L, 0.80 ± 0.41  × 10^9^/L, and 19.89 ± 14.26 in patients with POF, and 9.46 ± 4.21  × 10^9^/L, 1.12 ± 0.49  × 10^9^/L, and 10.80 ± 9.22 in patients without POF (all *P* < 0.001). Patients who spent more than 7 days in ICU also demonstrated a higher neutrophil count (12.61 ± 5.19 vs 9.61 ± 4.44  × 10^9^/L; *P* < 0.001), NLR (19.24 ± 13.70 vs 11.21 ± 9.86; P < 0.001), and a lower value of lymphocyte count (0.81 ± 0.40 vs 1.11 ± 0.50  × 10^9^/L; *P* < 0.001). Significant difference was found in the level of admission neutrophils (12.83 ± 6.72 vs 10.06 ± 4.57  × 10^9^/L), lymphocytes (0.61 ± 0.29 vs 1.07 ± 0.49  × 10^9^/L), and NLR (23.81 ± 15.09 vs 12.19 ± 10.59) between survivors and nonsurvivors (all *P* < 0.001).

### 3.4 Predictive value of neutrophil, lymphocyte, and NLR

**Figure 2:**
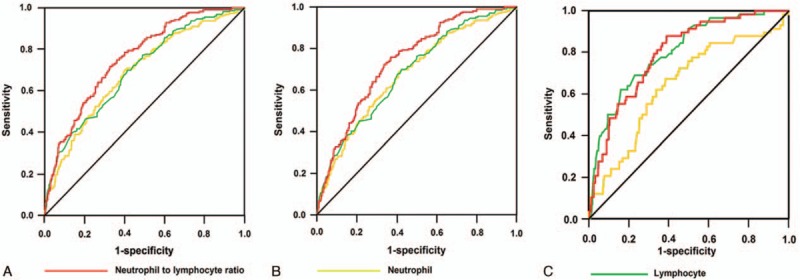
Receiver operated characteristic curve of neutrophil, lymphocyte count, and NLR for the diagnosis of POF (A), ICU stay >7 days (B), and in-hospital mortality (C). ICU = intensive care unit, NLR = neutrophil to lymphocyte ratio, POF = persistent organ failure.

ROC analysis was utilized to assess the diagnostic performance of neutrophil, lymphocyte, and NLR detected on admission for POF, ICU stay >7 days, and in-hospital mortality (Fig. 2). The NLR and lymphocyte count had a similar predictive performance for in-hospital mortality (AUC 0.79, 95% CI 0.74–0.85 vs AUC 0.80, 95% CI 0.75–0.86; *P* > 0.05), which was better than that of neutrophil count (AUC 0.65, 95% CI 0.57–0.72; *P* < 0.05). However, the NLR had a superior predictive performance than lymphocytes and neutrophils for POF (AUC 0.76, 95% CI 0.72–0.79 vs AUC 0.70, 95% CI 0.66–0.74 and AUC 0.69, 95% CI 0.65–0.73; *P* < 0.05) and ICU stay >7 days (AUC 0.74, 95% CI 0.71–0.78 vs AUC 0.68, 95% CI 0.64–0.72 and AUC 0.67, 95% CI 0.63–0.72; *P* < 0.05). The best NLR cutoffs for diagnosing POF, ICU stay >7 days, and in-hospital mortality were 11.45, 10.56, and 11.44, respectively. We decided to choose NLR = 11 as the cutoff value. Using the cutoff of 11, the sensitivity of NLR for the prediction of POF was 72%, the specificity was 67%, the PPV was 39%, and the NPV was 89%. For the diagnosis of ICU stay >7 days, the sensitivity was 76%, the specificity was 63%, the PPV was 35%, and the NPV was 91%. For predicting in-hospital mortality, the sensitivity, specificity, PPV, and NPV were 88%, 61%, 13%, and 99%, respectively.

### 3.5 Association between neutrophil, lymphocyte, NLR, and outcomes

Clinical characteristics of patients according to NLR levels (cutoff: 11) are presented in Table 2. When AP patients were stratified according to the higher normality cutoff of NLR (cutoff: 11), the age of AP patients with NLR ≥ 11 were much older, and they were more likely to present with preexisting comorbidities (including chronic respiratory disease, chronic renal disease, and cardiovascular disease). Furthermore, patients with NLR ≥ 11 showed significantly higher levels of hematocrit, glucose, creatinine, fibrinogen, and total leukocyte count, while platelet count, levels of albumin, sodium, and calcium were statistically lower. No significance was observed in respect to gender, drinking and smoking habit, and serum potassium level between patients with and without NLR ≥ 11. Taking NLR ≥ 11 as a dichotomous variable, the incidence of POF, ICU stay >7 days, and in-hospital mortality was 38.1%, 34.8%, and 12.0% in patients with NLR ≥ 11, and 11.1%, 9.8%, and 1.3% in patients with NLR < 11 (all *P* < 0.001).

**Table 2:**
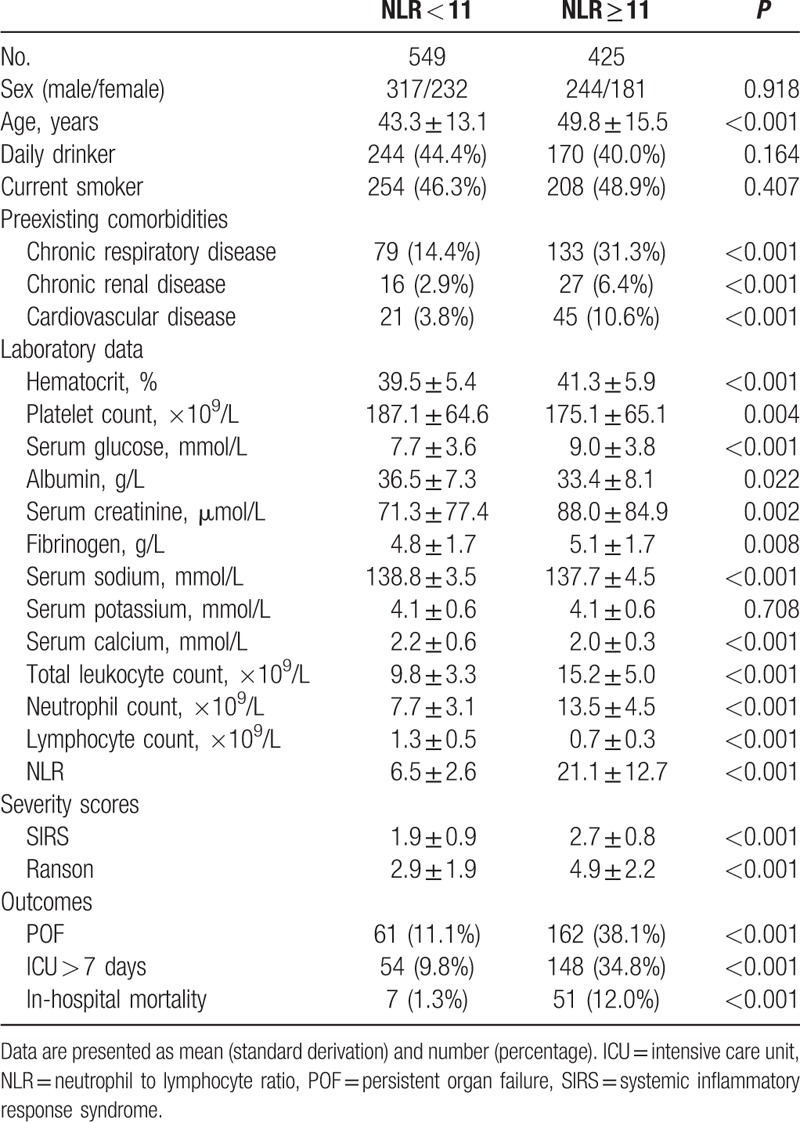
Clinical characteristics of patients according to neutrophil to lymphocyte ratio (cutoff value:11).

Table 3 demonstrates HRs and 95% CI for POF, ICU stay >7 days, and in-hospital mortality according to NLR levels (cutoff: 11), neutrophil count (presence or absence of neutrophilia), and lymphocyte count (presence or absence of lymphocytopenia). In univariate analysis, NLR ≥ 11, presence of neutrophilia, and lymphocytopenia were significantly associated with the incidence of outcomes. These trends remained significantly different even after adjusting for sex and age. After multivariate adjustment for confounding factors by analysis of covariance, for NLR ≥ 11, the trends remained statistically significant for POF (adjusted HR 1.37, 95% CI 1.00–1.89), ICU stay >7 days (adjusted HR 1.44, 95% CI 1.03–2.00), and in-hospital mortality (adjusted HR 2.75, 95% CI 1.12–6.76) (all *P* < 0.001). Lymphocytopenia remained statistically associated with incidence of POF and in-hospital mortality. However, neutrophilia was not independently associated with the outcomes after multivariate adjustment.

**Table 3:**
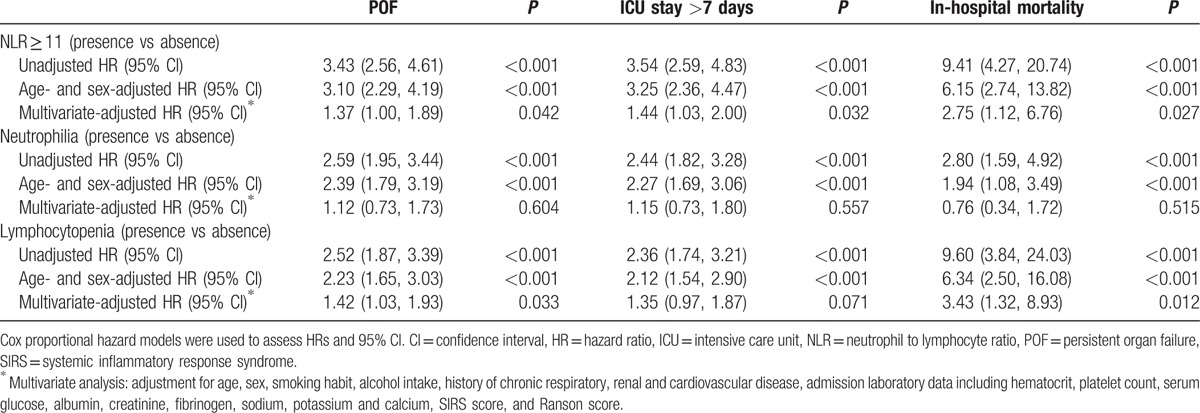
Risk of POF, ICU stay >7 days, and in-hospital mortality by NLR ≥ 11, neutrophilia, and lymphocytopenia.

Table 4 shows clinical features of the 974 patients according to quartiles of NLR. Age, levels of hematocrit, serum glucose, creatinine, fibrinogen, potassium, counts of total leukocyte, neutrophil, disease severity scores (SIRS and Ranson), and the frequency of preexisting comorbidities increased, whereas levels of platelet count, albumin, sodium, calcium, and counts of lymphocyte decreased with higher NLR quartiles. When AP patients were stratified according to NLR quartiles, there was a statistically significant stepwise increase in the incidence of POF (4.1%, 14.8%, 27.2%, and 45.5% for quartile 1–4; *P* < 0.001), ICU stay >7 days (3.7%, 14.0%, 25.8%, and 39.3% for quartile 1–4; *P* < 0.001), and in-hospital mortality (0.8%, 1.6%, 7.4%, and 13.9% for quartile 1–4; *P* < 0.001) with increasing quartile of baseline NLR.

**Table 4:**
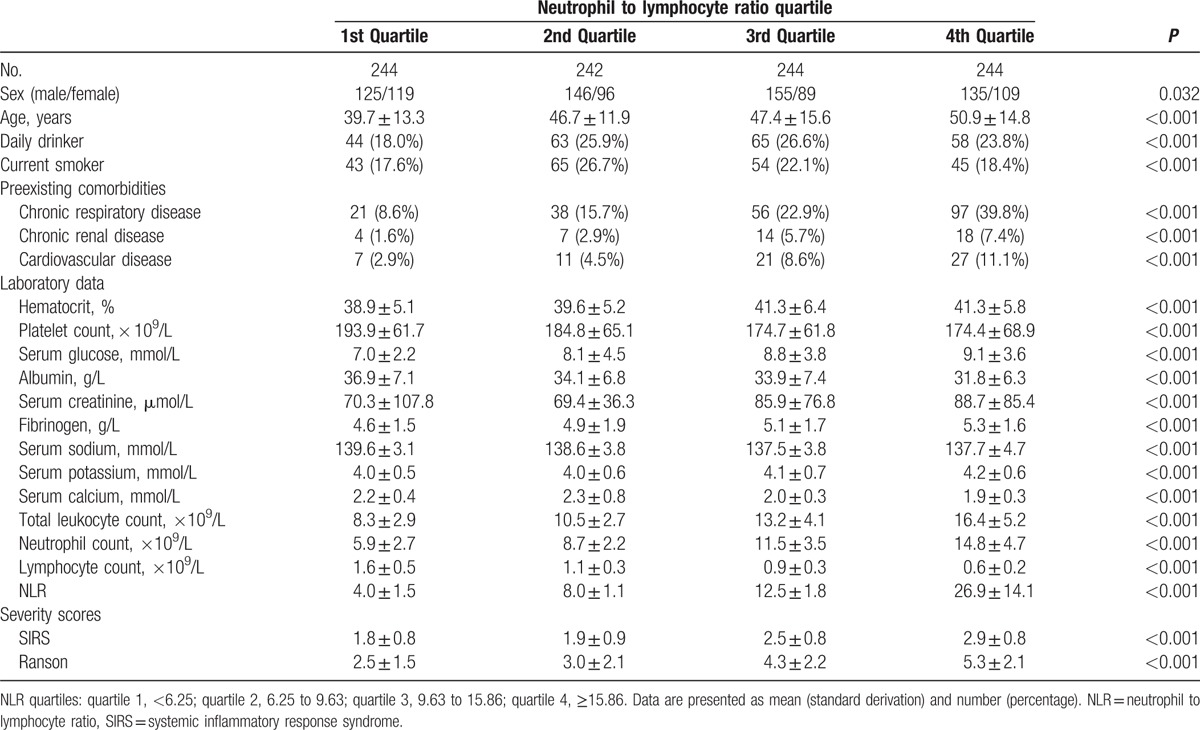
Clinical features of patients by quartiles of neutrophil to lymphocyte ratio.

Table 5 shows HRs and 95% CIs for POF, ICU stay >7 days, and in-hospital mortality by quartile of NLR with quartile 1 (lowest quartile) as reference. There was a positive trend for the association across increasing NLR quartiles and incidence of POF, ICU stay >7 days, and in-hospital mortality, *P* values for trends across quartiles were all <0.001. These trends remained significantly different even after adjusting for sex and age. After multivariate adjustment, the *P* values for trends across quartiles remained statistically significant (*P* = 0.007 for POF, *P* = 0.016 for ICU stay >7 days, and *P* = 0.028 for in-hospital mortality). The adjusted HRs for highest NLR versus the lowest quartile were 2.80 (95% CI 1.42–5.51) (POF), 2.79 (95% CI 1.37–5.70) (ICU stay >7 days), and 2.22 (95% CI 0.49–10.05) (in-hospital mortality).

**Table 5:**
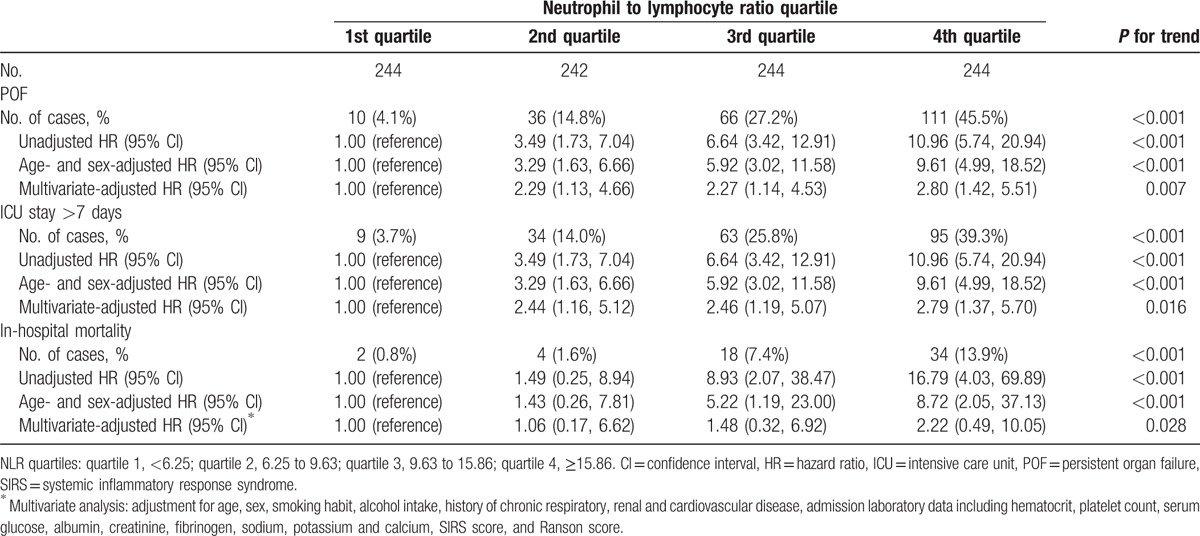
HRs for POF, ICU stay >7 days, and in-hospital mortality by neutrophil to lymphocyte ratio quartile.

We next evaluated HRs and 95% CI for POF, ICU stay >7 days, and in-hospital mortality by quartile of neutrophil count and lymphocyte count with quartile 1 (lowest quartile) as the reference quartile (Supplementary Table 1 and Supplementary Table 2). When AP patients were stratified according to neutrophil quartiles, there was a statistically significant stepwise increase in the incidence rate of POF, ICU stay >7 days, and in-hospital mortality with increasing quartile of neutrophils. However, after multivariate adjustment, no significant association between each outcome and quartiles of neutrophils was found (Supplementary Table 1). When AP patients were stratified according to lymphocyte quartiles, there was a statistically significant stepwise decrease in the incidence rate of POF, ICU stay >7 days, and in-hospital mortality with increasing quartile of lymphocytes. For admission lymphocyte count, after multivariate adjustment, the *P* values for trends across quartiles remained statistically significant (*P* = 0.005 for POF, *P* = 0.016 for ICU stay more than 7 days, and *P* < 0.001 for in-hospital mortality). Lymphocyte count quartiles were inversely associated with POF (adjusted HR for highest quartile 0.67, 95% CI 0.41–1.09), ICU stay >7 days (adjusted HR for highest quartile 0.74, 95% CI 0.44–1.22), and in-hospital mortality (adjusted HR for highest quartile 0.23, 95% CI 0.05–0.98) (Supplementary Table 2).

## 4 Discussion

In the present research, we examined the involvement of NLR at admission with incidence of POF, longer ICU stay, and in-hospital mortality in an Asian Chinese population of patients with AP.

The results revealed significant involvement of NLR in all outcomes even after adjustment for relevant potential confounding factors, indicating that higher NLR is an independent prognostic factor for the outcomes in AP.

The finding suggests that elevated number of neutrophil accompanied by decreased number of lymphocyte might play a role in the development and progression of AP.

NLR is first reported as an easily measurable parameter of systemic inflammation and stress by Zahorec^[10]^ in critically ill. Since then, its prognostic value in numerous clinical conditions has been clarified. Three previous studies revealed an association between worse outcomes and elevated NLR in patients with pancreatic cancer.^[11–13]^ More investigations of systemic inflammation-based NLR value may identify novel treatment strategies for patients with cancer.^[14]^ One recent study by Salciccioli et al^[15]^ found an association between NLR and mortality in a population of unselected critically ill patients.

With respect to the relationship between NLR and systemic inflammatory response, 4 studies, including ours, have recently investigated the relationships between NLR and outcome in a population of patients with AP. Utility of the NLR in AP was first investigated by Azab et al.^[8]^ They noticed that the NLR was a more sensitive parameter than total leukocyte counts in predicting ICU admission and longer hospitalization. They suggested an NLR cutoff value of >4.7 as a warning sign of poor outcome. But the in-hospital mortality was low (1.4%). Besides, they failed to assess records of PNec and OF, 2 major factors associated with mortality. What is more, information regarding the time between symptom onset and admission to the hospital in individuals was not available. Suppiah et al^[9]^ later revealed that NLR measured during the first 48 hours of hospitalization was significantly associated with the risk of developing severe form of AP (classified by 1992 Atlanta criteria). But the study was confined to a limited number of patients (n = 146). Most patients (84.9%) were diagnosed as mild AP without local or systemic complications and organ dysfunction. And the time from abdominal pain onset to hospitalization remained unclear.^[16]^ Recently, Gulen et al^[17]^ investigated the association between NLR and early mortality (within 48 hours) in patients with nontraumatic AP. They suggested NLR was not an independent prognostic factor.

However, no previous study has investigated the association between admission NLR and increased incidence of POF, longer ICU stay, and in-hospital mortality in AP. Therefore, to our knowledge, this study is the first time to show that elevated NLR is significantly associated with increased risk of adverse outcome in an Asian Chinese population with AP.

AP is an inflammatory featured by activation of both innate and adaptive immune systems. The former is directed by macrophages and neutrophils with secretion of proinflammatory chemokines and cytokines (such as interleukin-1β and tumor necrosis factor-α), granule enzymes, and reactive oxygen species. The activation of neutrophils is vital for host defense in SIRS, but their overzealous recruitment leads to massive cell transmigration to the site of inflammation, and their subsequent activation results in the release of aggressive defense molecules, leading to relevant tissue destruction and organ dysfunction.^[4,18–20]^ Mainly composed of lymphocytes, the latter is activated secondary to cytokine cascade and mediates the subsequent regulation of inflammatory response.^[21–23]^ Uncontrollable inflammation response corresponds with SIRS, OF, and POF, which are independently correlated with prolonged hospitalization, high rates of morbidity, and mortality.^[24–27]^ It is well established that the acute systemic inflammatory response is associated with alterations in peripheral leukocyte, particularly the presence of neutrophilia with a relative lymphocytopenia (elevated NLR), reflecting the acute changes of the immune system in the setting of AP.

The relationship between neutrophils, lymphocytes, and NLR and POF, in-hospital mortality in AP has not been studied yet. In our stratified analyses, we show here for the first time that NLR is an independent predictive factor of POF, ICU stay >7 days, and in-hospital mortality, and its predictive value is significantly higher than other differential parameters. Using NLR ≥ 11 as a dichotomous variable, the trends remained statistically significant for POF, ICU stay >7 days, and in-hospital mortality. When patients were stratified according to NLR quartiles, there was a statistically significant stepwise increase in the incidence of the 3 outcomes. After multivariate adjustment, the *P* values for trends across quartiles remained statistically significant.

Several limitations are evident in our study. First, as a retrospective study, we did not have sufficient power to assess the overall mortality rate. In our study, the in-hospital mortality was relatively lower. The causality role of admission NLR in the mortality of AP, however, requires to be investigated further in a prospective validation study. Second, our study population was captured at a tertiary care center. This is reflected by the high proportion of patients with POF. This selection bias would overestimate the risk of higher NLR for the outcomes. Besides, the retrospective collected data did not allow us to evaluate follow-up information such as the incidence of infected pancreatic necrosis, a major cause of mortality in the late phase of AP. In addition, we examined one-time measurement of clinical data and single measurement could lead to the misclassification of exposure, confounders, disease severity, and outcomes. Finally, patients included in our study were all Asian Chinese. As race and ethnicity may affect variance in the distribution of differential leukocyte, the results may not be generalized in other racial population.

There are also several strengths of the present study. First, to the best of our knowledge, this is the 1st detailed study of the impact of NLR on incidence of POF, longer ICU stay, and in-hospital mortality. Second, we evaluated a large population of AP patients with sufficient available data, including various clinical outcomes from a single institution. Third, we only include patients who admitted to our center within 72 hours from AP symptom onset and without any medical treatment. It is possible that the use of medication may rapidly influence the distribution of peripheral leukocytes.

In conclusion, our study demonstrates for the first time that increased NLR determined upon admission is associated with incidence of POF, ICU stay >7 days, and in-hospital mortality in an Asian Chinese population with AP. We suggest that prospective research is needed to clarify the potential mechanisms of these associations.

## Acknowledgments

The authors thank grants from the National Natural Science Foundation Program of China (No. 02.07.13020062) or the support. The authors also thank the staff from Pancreatic Disease Institute of Union Hospital (Wuhan, China) for their support, and Dr Changzhong Chen and Dr Xinglin Chen from EmpowerStats for the assistance of data analysis and illustration.

## Supplementary Material

Supplemental Digital Content
