# Supplementary material for: Neutrophil to lymphocyte ratio predicts persistent organ failure and in-hospital mortality in an Asian Chinese population of acute pancreatitis
Source: Medicine (Baltimore). 2016 Sep 16;95(37):e4746. doi: 10.1097/MD.0000000000004746 (PMC5402566; doi:10.1097/MD.0000000000004746)
Supplement: Supplemental Digital Content [file medi-95-e4746-s001.doc]

**SUPPLEMENTARY TABLE 1. HRs for POF, ICU stay > 7 days and in-hospital mortality by neutrophil quartile**

|  | Neutrophil quartile | | | |  |
| --- | --- | --- | --- | --- | --- |
|  | **1st Quartile** | **2nd Quartile** | **3rd Quartile** | **4th Quartile** | ***P*-value for trend** |
| **No.** | 244 | 238 | 247 | 245 |  |
|  |  |  |  |  |  |
| **POF** |  |  |  |  |  |
| **No. of cases (%)** | 23 ( 9.4%) | 42 (17.6%) | 60 (24.3%) | 98 (40.0%) | <0.001 |
| **Unadjusted HR (95% CI)** | 1.00 (reference) | 1.87 (1.13, 3.11) | 2.58 (1.59, 4.17) | 4.24 (2.69, 6.68) | <0.001 |
| **Age- and sex-adjusted HR (95% CI)** | 1.00 (reference) | 1.71 (1.03, 2.86) | 2.23 (1.37, 3.63) | 3.77 (2.38, 5.96) | <0.001 |
| **Multivariate-adjusted HR (95% CI)** | 1.00 (reference) | 1.23 (0.73, 2.08) | 1.42 (0.87, 2.32) | 1.40 (0.87, 2.25) | 0.165 |
|  |  |  |  |  |  |
| **ICU stay > 7 days** |  |  |  |  |  |
| **No. of cases (%)** | 21 ( 8.6%) | 40 (16.8%) | 52 (21.0%) | 89 (36.3%) | <0.001 |
| **Unadjusted HR (95% CI)** | 1.00 (reference) | 1.95 (1.15, 3.31) | 2.45 (1.47, 4.06) | 4.22 (2.62, 6.79) | <0.001 |
| **Age- and sex-adjusted HR (95% CI)** | 1.00 (reference) | 1.79 (1.05, 3.05) | 2.14 (1.28, 3.57) | 3.78 (2.34, 6.11) | <0.001 |
| **Multivariate-adjusted HR (95% CI)** | 1.00 (reference) | 1.35 (0.78, 2.32) | 1.38 (0.82, 2.32) | 1.41 (0.86, 2.71) | 0.251 |
|  |  |  |  |  |  |
| **In-hospital mortality** |  |  |  |  |  |
| **No. of cases (%)** | 7 ( 2.9%) | 9 ( 3.8%) | 18 ( 7.3%) | 24 (9.8%) | <0.001 |
| **Unadjusted HR (95% CI)** | 1.00 (reference) | 1.32 (0.49, 3.54) | 2.54 (1.06, 6.08) | 3.41 (1.47, 7.92) | <0.001 |
| **Age- and sex-adjusted HR (95% CI)** | 1.00 (reference) | 1.13 (0.42, 3.04) | 1.52 (0.62, 3.72) | 2.18 (0.93, 5.15) | <0.001 |
| **Multivariate-adjusted HR (95% CI)*** | 1.00 (reference) | 0.84 (0.28, 2.50) | 0.99 (0.40, 2.46) | 0.83 (0.34, 2.02) | 0.735 |

Neutrophil quartiles: quartile 1, <6.89 ×10^9^/L; quartile 2, 6.89 - 9.60 ×10^9^/L; quartile 3, 9.60 - 12.67 ×10^9^/L; quartile 4, ≥12.67 ×10^9^/L.

* Multivariate analysis: adjustment for age, sex, smoking habit, alcohol intake, history of chronic respiratory, renal and cardiovascular disease, admission laboratory data including hematocrit, platelet count, serum glucose, albumin, creatinine, fibrinogen, sodium, potassium and calcium, SIRS score, and Ranson score.

Abbreviations: CI, confident interval; HR, hazard ratio; ICU, intensive care unit; POF, persistent organ failure; SIRS, systemic inflammatory response syndrome.

**SUPPLEMENTARY TABLE 2. HRs for POF, ICU stay > 7 days and in-hospital mortality by lymphocyte quartile**

|  | Lymphocyte quartile | | | |  |
| --- | --- | --- | --- | --- | --- |
|  | **1st Quartile** | **2nd Quartile** | **3rd Quartile** | **4th Quartile** | ***P*-value for trend** |
| **No.** | 246 | 248 | 256 | 224 |  |
|  |  |  |  |  |  |
| **POF** |  |  |  |  |  |
| **No. of cases (%)** | 93 (41.5%) | 63 (24.6%) | 45 (18.1%) | 22 ( 8.9%) | <0.001 |
| **Unadjusted HR (95% CI)** | 1.00 (reference) | 0.59 (0.43, 0.82) | 0.44 (0.31, 0.62) | 0.22 (0.14, 0.34) | <0.001 |
| **Age- and sex-adjusted HR (95% CI)** | 1.00 (reference) | 0.62 (0.45, 0.86) | 0.48 (0.34, 0.69) | 0.25 (0.16, 0.40) | <0.001 |
| **Multivariate-adjusted HR (95% CI)** | 1.00 (reference) | 0.95 (0.68, 1.32) | 0.67 (0.46, 0.98) | 0.67 (0.41, 1.09) | 0.005 |
|  |  |  |  |  |  |
| **ICU stay > 7 days** |  |  |  |  |  |
| **No. of cases (%)** | 81 (36.2%) | 60 (23.4%) | 40 (16.1%) | 21 (8.5%) | <0.001 |
| **Unadjusted HR (95% CI)** | 1.00 (reference) | 0.65 (0.46, 0.91) | 0.45 (0.31, 0.65) | 0.24 (0.15, 0.38) | <0.001 |
| **Age- and sex-adjusted HR (95% CI)** | 1.00 (reference) | 0.68 (0.48, 0.95) | 0.49 (0.33, 0.72) | 0.27 (0.17, 0.44) | <0.001 |
| **Multivariate-adjusted HR (95% CI)** | 1.00 (reference) | 1.06 (0.75, 1.50) | 0.72 (0.48, 1.06) | 0.74 (0.44, 1.22) | 0.016 |
|  |  |  |  |  |  |
| **In-hospital mortality** |  |  |  |  |  |
| **No. of cases (%)** | 37 (16.5%) | 12 (4.7%) | 7 (2.8%) | 2 (0.8%) | <0.001 |
| **Unadjusted HR (95% CI)** | 1.00 (reference) | 0.28 (0.15, 0.54) | 0.17 (0.07, 0.38) | 0.05 (0.01, 0.20) | <0.001 |
| **Age- and sex-adjusted HR (95% CI)** | 1.00 (reference) | 0.35 (0.18, 0.68) | 0.26 (0.11, 0.58) | 0.09 (0.02, 0.37) | <0.001 |
| **Multivariate-adjusted HR (95% CI)*** | 1.00 (reference) | 0.40 (0.20, 0.82) | 0.29 (0.11, 0.75) | 0.23 (0.05, 0.98) | <0.001 |

Lymphocyte quartiles: quartile 1, <0.70 ×10^9^/L; quartile 2, 0.70 - 0.94 ×10^9^/L; quartile 3, 0.94 - 1.31 ×10^9^/L; quartile 4, ≥1.31 ×10^9^/L.

* Multivariate analysis: adjustment for age, sex, smoking habit, alcohol intake, history of chronic respiratory, renal and cardiovascular disease, admission laboratory data including hematocrit, platelet count, serum glucose, albumin, creatinine, fibrinogen, sodium, potassium and calcium, SIRS score, and Ranson score.

Abbreviations: CI, confident interval; HR, hazard ratio; ICU, intensive care unit; POF, persistent organ failure.
